# Population affinity estimation in forensic anthropology: a South African perspective

**DOI:** 10.1007/s00414-025-03529-8

**Published:** 2025-05-26

**Authors:** Thandolwethu Mbonani, Ericka L’Abbé, Ding-Geng Chen, Alison Ridel

**Affiliations:** 1 Department of Anatomy, University of Pretoria, Pretoria, South Africa; 2 Department of Statistics, University of Pretoria, Pretoria, South Africa

**Keywords:** Morpho-phenotypic traits, Population affinity, Forensic anthropology, South African population, Forensic identification

## Abstract

**Abstract:**

Forensic anthropologists face the complex task of estimating population affinity from skeletal remains, a process that involves inferring culturally constructed “social race” from biological tissues, a challenge further complicated by the nuanced distinction between population affinity and “race”. The difficulty in making these estimations arises from the complex interplay between social constructs of race, skeletal morphology, and geographic origin. These factors are further influenced by elements such as assortative mating and institutional racism in regions such as South Africa and the United States. The interaction between cultural factors and biological traits raises the question of whether the challenges in estimating population affinity are inevitable or due to a limited understanding of human variation. To address this knowledge gap, this paper presents a review of population affinity estimation in forensic anthropology, with a focus on the South African context. It provides foundational background and historical insights, explores the medico-legal significance of population affinity, and critically evaluates both traditional and emerging estimation methods. By highlighting regional challenges and recent advancements, this review aims to enhance understanding and contribute to ongoing debates in the field.

**Clinical trial number:**

Not applicable.

## Introduction

Population affinity estimation is a critical component of forensic anthropology, providing valuable insights into an individual’s likely social group based on skeletal morphology [1, 2]. This process is essential for constructing biological profiles used in medico-legal investigations, mass disasters, and historical identifications, while also informing culturally appropriate repatriation efforts and contributing to broader understandings of human variation [1–3].

Historically, this process relied on typological models that rigidly classified humans into discrete racial categories based on observable traits such as skin colour, cranial morphology, body proportions, and behaviour. These models, grounded in 18th- and 19th-century frameworks, oversimplified human variation and reinforced biased hierarchical constructs [4–8]. Over the past century, however, genetic and anthropological research have prompted a shift toward population-based approaches that recognise the continuous and complex nature of biological variation, as shaped by evolutionary, environmental, and sociocultural processes [3, 9–11]. For an in-depth view of this historical development, see Ousley et al. [12], Brace [13], and DiGangi and Hefner [9].

The ANSI/ASB Standard 132, established by the American Academy of Forensic Sciences Standards Board, formalizes this approach to population affinity estimation by defining it as the probabilistic assessment of whether skeletal traits align with known reference groups [14]. This standard discourages race-based typologies and emphasizes the use of validated metric and non-metric methods, ethical practice, and context-sensitive reference data [3]. These principles are particularly important in South Africa, where population history, shaped by admixture, migration, and apartheid, presents unique challenges for forensic practitioners [15, 16]. Although the global literature on population affinity estimation is extensive, there remains a significant gap in understanding how these methods apply to regions with complex demographic histories, such as South Africa. Issues such as trait overlap, limited skeletal collections, and resource constraints highlight the limitations of global methods and underscore the need for localized standards and more comprehensive reference datasets tailored to the South African context.

This review briefly traces the shift from raced-based typologies to population affinity estimation, and then critically examines both the traditional and modern methods and technologies used globally. It focusses especially on their applicability in the South African context, highlighting region-specific challenges, ethical considerations, and the need for local reference data to improve forensic accuracy and inclusivity.

## Population affinity in forensic anthropology

Forensic anthropology continues to navigate the tension between outdated racial concepts and contemporary understandings of human biological variation. Despite the scientific discrediting of ‘race” as a biological construct [10, 17], it continues to shape public perception and influence forensic practice. Early calls to replace “race” with “ancestry” [10] and more recent efforts to adopt “population affinity” [3] reflect an ongoing shift toward terminology and methods grounded in evolutionary theory and population history. However, this transition remains incomplete, and population affinity is still underutilized in standard forensic practice and literature [3, 11, 18, 19].

### Historical shifts in terminology and frameworks

The classification of humans into distinct groups has deep historical roots. Early typologies—from Bernier’s and Linnaeus’ classifications to Blumenbach’s five races—linked physical traits to presumed behavioural and moral qualities [1, 13, 20], providing pseudo-scientific justification for colonialism, slavery, and systematic racism [21]. Though such models were later undermined by Darwinian evolution and the work of anthropologists such as Franz Boas [9, 10, 22], typological thinking persisted well into the 20th century.

Even as anthropologists began recognising the influence of the environment, migration, and gene flow on human variation [23–25], physical anthropology heavily relied on biased skeleton collections primarily compromising of White, African-American, and Asian individuals, and terminology reflecting United States(US) Census categories [20, 26, 27]. In South Africa, while political and ethical constraints limited the development of similar classification frameworks, collections such as the Pretoria Bone and Raymond A. Dart collections, along with the country’s population diversity, have supported research on morphological variation [27].

Today, the majority of genetic variation is understood to occur within populations rather than between them [1], and rigid racial categories are widely criticized as both scientifically flawed and socially harmful [28, 29]. For deeper historical contexts, see Cunha and Ubelaker [1], Chrysostomou and Thompson [20], and Gill [21].

### From “race” and “ancestry” to population affinity

From the1960s onward, anthropologists such as Brace and Livingstone [30, 31] challenged the biological validity of race, demonstrating that physical traits vary independently across populations and do not form consistent, biologically discrete clusters. While the term “ancestry” gained traction in the 1990s, its use often failed to depart fully from typological models [3, 32, 33].

Genomic research in the early 2000s further highlighted the limitations of “race” and “ancestry” as biological categories, emphasizing the value of population-based interpretations rooted in evolutionary and environmental contexts [3, 34]. Nonetheless, forensic tools such as 3D-ID and AncesTrees sometimes reinforced continental or racial groupings (e.g., African, Asian, European), raising concerns about accuracy and ethical implications [35–38].

The emerging concept of population affinity seeks to address these limitations by focusing probabilistic estimation of group membership based on morphological or genetic similarity, while explicitly incorporating evolutionary theory, population history, and regional specificity [3, 11, 25, 39, 40]. This approach is particularly suited to contexts such as the US or South Africa, where historical factors—such as miscegenation laws, systemic racism, and patterns of assortative mating—have shaped population structures [15, 16, 41].

Future directions in forensic anthropology require the consistent use of precise terminology, transparent definitions, and context-sensitive methods. Embracing population affinity allows for a more scientifically accurate and ethically responsible approach to human identification [3, 11, 42–44].

### The significance of population affinity estimation

Bethard and DiGangi [45] sparked renewed debate on the ethical and practical challenges of estimating population affinity in forensic anthropology. While race is widely accepted as a social construct, medicolegal systems still require racial classification of skeletal remains [46]. Although most anthropologists reject the notion of biological race [46, 47], assigning a “social race” remains central to identification, as missing persons are typically described using racialised terms [1, 10, 20, 46]. Cunha and Ubelaker [1] emphasize using socially familiar terminology to facilitate identifications, while avoiding outdated racial models. This reflects a broader tension in the field: addressing a legacy of scientific racism while meeting the needs of justice systems [46].

Population affinity estimation—used to infer a likely “social race” from skeletal features—remains a key tool for linking unidentified remains to missing persons reports [20], especially in settings such as the US and South Africa, where such classifications carry social and legal weight. Nonetheless, practitioners must remain cautious to avoid reinforcing harmful racial stereotypes through the misuse of biological classifications. As a result, this practice demands ongoing reflection, recognition of bias, and careful application of socially constructed categories.

Although “race” fails to capture the full scope of human biological variation, skeletal traits often reflect regional population patterns shaped by history, migration, language, and nationality [10, 46, 48]. These patterns allow for probabilistic assessments of likely group membership, supporting identification efforts through a more nuanced, gradient-based understanding of human variation [46]. Advances in methodology, including the use of global reference samples and probabilistic methods, have improved classification accuracies—exceeding 90% in certain cases [35]—highlighting the practical value of population affinity estimation. Despite ongoing controversy, including population affinity in the biological profile remains essential, as it reflects the societal and institutional role of race in shaping identification practices. Forensic anthropologists must balance the practical need for affinity estimation in investigations and repatriations with careful consideration of its ethical, social, and historical implications [49]. Ongoing dialogue is essential to ensure this practice remains both scientifically rigorous and socially responsible.

## Methods used for estimating population affinity

Population affinity estimation in forensic anthropology draws on cranial, dental, and postcranial analyses, with cranial features—especially the mid-face—often considered most informative [1, 20, 40, 50]. Two main approaches are used: metric and non-metric. Metric methods involve quantitative skeletal measurements analysed statistically to estimate group membership, promoting objectivity and reproducibility [40]. Non-metric methods, in contrast, rely on visual assessment of morphological traits, offering nuanced insights but limited by subjectivity and observer experience [1]. Combining both approaches allows for a more comprehensive evaluation [20, 51]. However, accurate estimations require population-specific validation, as overlapping morphological variation, further compounded by increasing global admixture complicates classification, particularly in the absence of genomic data.

This section provides a general overview of the methodologies commonly employed in forensic anthropology for assessing population affinity. It outlines widely used tools and software developed internationally, focusing on their methodological frameworks, applications, and limitations. While many of these tools have not yet been systematically applied or validated within the South African context, understanding them is essential for situating this study within the broader global discourse on forensic population affinity estimation.

### Metric approaches

#### Cranial

Craniometry, based on defined cranial landmarks and inter-landmark distances (ILDs), has long served as a central method in forensic anthropology due to its objectivity and reproducibility [5, 13, 52]. Introduced in the 19th century, its applications shifted from typological classification to probabilistic group differentiation. By the 1960s, discriminant function analysis (DFA) was integrated to improve population-level comparisons, though it had limitations in misclassifying underrepresented groups [20, 53]. Modern practice follows standardized protocols developed for forensic skeletal analysis [54, 55], with biodistance analysis continuing to demonstrate value in both population history and forensic science [56].

FORDISC is a widely used tool that applies DFA to cranial and postcranial measurements [50]. It compares skeletal measurements of unknown individuals to data in the Forensic Anthropology Databank (FDB), which includes over 16,000 individuals. The FDB includes reference populations for craniometric measurements (see Table 1), with more comprehensive data for cranial measurements than postcranial. Postcranial data is available for African, White, and Hispanic populations, with sex data varying across groups. The accuracy of FORDISC depends on the quality and representativeness of its reference data, which is largely North American. This can limit its reliability for individuals from underrepresented or admixed populations [56]. For instance, the label “Hispanic” is culturally defined and not biologically uniform, making it inappropriate for individuals such as Portuguese, despite potential geographic assumptions [50]. This underscores the importance of critical evaluation when applying classification systems across diverse populations.

**Table 1:** Distribution of reference groups for cranial data on FORDISC

Forensic Anthropology Databank			
Reference population	Females	Males	*n*
African Americans	137	224	361
White Americans	454	737	1191
Native Americans	32	59	91
Hispanic Americans	74	281	355
Japanese	58	84	142
Chinese	-	80	80
Guatemalan	-	83	83
Vietnamese	-	51	51
**n**	755	1599	2354

Other tools such as CRANID, AncesTrees, and (hu)MANid have emerged to complement or refine population affinity estimation. CRANID compares 29 cranial variables against a global database of over 3,000 individuals. It uses DFA and nearest neighbour analyses for classification [57, 58]. Validation studies show moderate accuracy rates of 39–48%, though performance improves with the inclusion of sex data and more comprehensive reference samples [58]. AncesTrees, a random forest-based tool that uses 23 cranial measurements from the Howells series [59], demonstrated moderate to high classification accuracy (ranging from 34.55 to 100%) for Southwestern European populations, with occasional misclassifications to nearby regions (e.g., Northern and Central Europe or Northeast Africa) [60]. Meanwhile, (hu)MANid, a web-based application, incorporates mandibular data and both metric and morphoscopic traits. In the initial validation study [61], (hu)MANid reported 84% accuracy for sex prediction and 53% for population affinity using multivariate approaches.

These tools have shown utility in various regional contexts; however, their effectiveness largely hinges on the representativeness of the reference data and their applicability to specific populations. In the South African context, their promise is limited by the lack of regionally appropriate reference samples. A key limitation of DFA, for instance, is the extent to which the reference populations reflect the diversity of the target population. FORDISC, for example, is built primarily on North American datasets, which may not adequately capture global human variation—particularly for individuals of “mixed” affiliation or for casework conducted outside of North America. Additionally, DFA is sensitive to statistical issues such as outliers and multicollinearity, both of which can skew results and diminish predictive accuracy [53, 62]. The method also relies on several statistical assumptions, including multivariate normality and homogeneity of variances. Although minor deviations from these assumptions are generally tolerated, significant violations—such as unequal group variances—can compromise classification performance. In such cases, alternative approaches such as logistic regression may provide more reliable results [63].

#### Postcranial

Postcranial methods remain underutilized compared to craniometric approaches, though recent studies, particularly involving the femur and tibia, have shown promising results [64]. As with craniometrics, reliance on simple indices should be avoided, as they can be influenced by factors such as mobility and physical stress [1].

Historically, postcranial analysis evolved alongside shifting anthropological and forensic interests [52]. Early work in the 19th and early 20th centuries by Broca [65], Davenport and Love [66], and Hrdlička [67] focused on physical differentiation between populations, often to categorize race, though these studies lacked standardized methods [50]. By the mid-20th century, attention shifted toward biomechanical and biodistance analyses, with region-specific traits gaining forensic relevance [68]. The Platymeric Index, for instance, linked femoral flattening to both locomotion and geographic patterns [69, 70], though subsequent studies questioned its broader applicability, especially among modern populations [71]. Other traits, such as the femoral curvature and intercondylar notch width, were also explored to differentiate African, White And Native American populations [72, 73], though issues with accuracy and observer reliability limited their forensic utility [74, 75].

Despite these limitations, research into the pelvis, tibia, cervical vertebrae, and hyoid bone continued into the 21st century [76–79], with some studies achieving high classification accuracy—up to 98%—in differentiating African and White American individuals [78, 79]. However, inconsistent protocols, poor interobserver reliability, and limited applicability to broader populations remain challenges [73, 80].

To improve utility, recent studies have turned to multivariate approaches such as DFA, combining multiple postcranial traits to enhance accuracy [81, 82]. Tools such as FORDISC reflect this shift but currently include postcranial data for only four groups: African and White American males and females [62, 83]. While these developments show progress, standardization and broader validation remain essential for effective forensic application across diverse populations.

#### Dental

Odontometrics—centred on mesiodistal, buccolingual, and cervical measurements measurement—has a long history, beginning with Muhlreiter’s work in 1874 [84], and standardised by Moorrees and Reed’s [85]. Tooth measurements are attractive due to their high preservation rate, genetic stability, and resistance to postmortem alteration [50, 86]. Emerging techniques such as polygonal shape analysis, geometric morphometrics, and enamel/dentin thickness measurements are being explored to capture population-specific variation [87–91].

Despite their potential, dental metrics remain underutilized in forensic contexts. Hanihara and Ishida [92] highlighted global dental variation patterns for population affinity estimation using open-access datasets. Pilloud et al. [93] applied DFA to this dataset [92], successfully classifying African, Asian, and European individuals, particularly when sex data was included. US-based studies by Kenyhercz et al. [90] and Harris and Foster [86] reported classification rates of 66–72% using intercusp distances and geometric morphometrics to distinguish African, White, and Hispanic American groups. Harris and Foster [86] demonstrated success with crown dimensions for population affinity and sex estimation, findings supported by Lease and Sciulli’s [94] work on deciduous teeth. Harris and Foster [86] further emphasized advantages of dental metrics, including excellent preservation, genetic stability, ease of data collection, and resistance to remodelling, making teeth valuable in long-term anthropological and forensic analyses.

### Non-metric approaches

Non-metric approaches in forensic anthropology are divided into epigenetic variants and morphoscopic traits. Epigenetic variants, or discrete traits, are dichotomous, non-pathological skeletal features (e.g., accessory bones, ossification anomalies) [55, 95] used in biodistance studies to assess biological relatedness [55]. In forensic applications, however, the traits used to assess population affinity may differ from traditional epigenetic variants due to the unique historical developmental of morphoscopic traits [96]. Morphoscopic traits refer to subtle variations in cranial morphology assessed through features such as bone shape, bony feature morphology, suture shape, presence or absence of specific features and trait prominence [96]. Historically assessed through subjective “trait lists” [29, 97, 98], morphoscopic analysis has since been systemized by Hefner [96, 99, 100], who with Ousley [101] introduced cranial macromorphoscopic (MMS) traits—quasi-continuous variables reflecting soft tissue variation [102, 103]. Standardization of cranial MMS traits has enhanced their objectivity and statistical reliability in forensic applications, supported by genetic associations [104]. In contrast, the postcranial skeleton has been less utilized [20]. Although elements such as the femur, pelvis, ribs, and vertebrae exhibit population-related morphological variation, their forensic application remains limited. The femur has seen the most use, but early studies [105–107] were largely descriptive and lacked statistical rigor [72]. More recent research has applied statistical frameworks and identified genetic components of postcranial traits [108], but practical use in forensic contexts remains secondary to cranial methods due to limited standardization and refinement.

#### Cranial

The use of cranial non-metric traits to estimate population affinity has its origins in the early 20th-centrury “trait list approach”, particularly Hooton’s Harvard list [33, 109, 110]. This method focused on morphologically visible, biologically inherited traits [109], and has continued to influence forensic practice through studies such as Rhine’s 1990 work [111]. However, though widely used, it remains controversial due to its subjectivity, observer dependency, and lack of empirical validation or error rate calculations [112].

To address these limitations, Hefner [96] introduced a standardized scoring protocol for MMS traits, improving replicability and aligning with standards for evidence in forensic science. MMS traits—standardised cranial features scored on ordinal and categorical scales—have demonstrated increasing classification accuracy across diverse populations [113–115], including Hispanic [116, 117], African, and African American groups [118, 119]. These applications demonstrate the growing reliability and versatility of cranial MMS traits in forensic anthropology. Inter- and intra-observer reliability has also improved with training and methodological refinement [115, 120].

Several tools support MMS data collection and analysis. The Macromorphoscopic Traits v1.61 was developed as part of the Macromorphoscopic Databank (MaMD) initiative [111], encompassing data from over 2,400 individuals, including historic Native American groups [121]. Osteoware, a broader data entry platform, also integrates Hefner’s [96] original 11 traits and additional features with supporting images and trait descriptions [111],

Statistical analysis of MMS traits typically employs nonparametric and machine learning approaches due to the categorical nature of the data [122]—including artificial neural networks (ANNs), random forest models (RFMs), support vector machines (SVMs), and k-nearest neighbors (k-NN)—frequently exceeding 85% classification accuracy in three-group comparisons [8, 59, 102, 111, 116, 123]. Other tools include OSSA (Optimized Summed Scored Attributes) [101]; effective for binary classifications (e.g., African vs. White American), the hefneR package for Bayesian modelling using 11 cranial MMS traits [96, 124], and the MaMD Analytical Tool, which employs an ANN to estimate population affinity and handle missing data using 10–16 MMS traits [125]. The integration of standardized MMS traits with such advanced analytical tools has significantly improved population affinity estimation in forensic anthropology. Nonetheless, continued validation across diverse populations and ethical implementation remain essential [126].

#### Postcranial

Historically underutilized due to concerns that environmental plasticity could obscure true ancestral differences [29, 127], postcranial non-metric traits have seen renewed forensic interest with the introduction of standardised scoring systems and machine learning applications [52]. Duray, Morter, and Smith [128] developed a scoring system for cervical vertebrae bifurcation to differentiate populations. Similarly, Finnegan and McGuire [129] applied six multivariate methods—including Bayesian analysis and DFA—to traditional postcranial traits, achieving classification accuracies of 53–95% in two-group comparisons. More recently, Spiros [130] standardized 11 postcranial traits, enhancing their forensic applicability. Spiros and Hefner [51] further improved classification by integrating cranial and postcranial data using models such as quadratic DFA, ANNs, RFMs, and SVMs. This combined approach increased classification accuracy by nearly 15% over single-region models. Their Combo MaMD Analytical software, employing an ANN for population classification, is now publicly available for forensic research [51]. Despite these advancements, current postcranial reference datasets remain limited—primarily representing African and White American groups—restricting broader applicability. To improve generalizability, further research is needed to expand population coverage and refine trait definitions and scoring for diverse and admixed populations [131].

#### Dental

Dental non-metric traits are particularly valuable in forensic anthropology due to their high heritability, preservation, and population-specific variation [132]. Traits such as incisor shovelling (common in Asian populations) and Carabelli’s cusp (more frequent in Europeans) have been consistently linked to broad population patterns, making them useful in both bioarchaeology and forensic contexts [132–135]. The standardization of non-metric dental trait assessment began with Dahlberg’s (1965) reference plaques [132], which formed the basis for modern systems such as the Arizona State University Dental Anthropology System (ASUDAS) [136]. ASUDAS includes 27 dental traits with standardized scoring [136] and has facilitated cross-population comparisons in large-scale anthropological studies [137–140].

Edgar [141] advanced the field by applying statistical models (e.g., logistic regression, Bayesian analysis) to estimate affinity based on dental traits, initially among African American and European populations, later including Hispanic groups [135]. In 2017, she published an illustrated guide to standardize these methods [142]. Irish [143] built on this by using 10 ASUDAS traits to differentiate among six major population groups, namely, Chinese Mongolian, European, Northern and Southern Native American, Polynesian, and sub-Saharan African, though the method lacked error rate reporting, limiting its forensic reliability [144]. More recently Scott et al. [145] introduced rASUDAS, a web-based tool using 21 dental traits and a Naive Bayes algorithm to estimate population affinity. Drawing from a database of ~ 30,000 individuals across seven regions, rASUDAS achieved up to 73% accuracy in three-group comparisons [145]. Though promising, it requires further validation, especially in forensic contexts. A notable application of rASUDAS was by Štamfelj et al. [146], who classified a 1500-year-old skeleton—likely a Hun warrior—into the East Asian group with 99.9% probability.

Human teeth are exceptionally durable and genetically informative, making them ideal for population affinity estimation, especially when other skeletal elements are damaged or absent [23]. However, due to overlapping trait frequencies across populations, dental data should be integrated with other lines of evidence to ensure accuracy [1].

### Other approaches

#### Geometric morphometrics

Geometric morphometrics (GMM) analyses shape variation by using Cartesian landmark coordinates and Procrustes analysis to remove size as a variable and enable direct comparison of shapes [16]. By reducing observer bias and increasing reproducibility, GMM offers clear advantages over traditional linear methods, particularly when applied to cranial elements such as the midface, which often show strong morphological differentiation [26, 147–149]. Two-dimensional(2D) and 3D GMM have shown classification accuracies exceeding 90% in distinguishing between groups such as African and European Americans [150]. Studies in Europe(e.g., Czech and French populations) similarly report high accuracies (~ 92%) when analysing cranial shape variations, though regional diversity must be considered for broader applicability [148]. Though the postcranial skeleton has traditionally been considered less reliable than the cranium for distinguishing population groups—due to its higher plasticity resulting from environmental influences [151]—recent GMM studies have achieved classification rates of 60–98% using the sacrum, humerus, and fibula [152, 153].

Notably, tools such as 3D-ID, a freely available software that enables the assessment of both population affinity and sex from cranial data, have increased GMM’s accessibility [154]. Additionally, elliptical Fourier analysis (EFA) is gaining traction for outline-based morphometrics [155]. This method quantifies the shape of objects by describing their outlines through a series of harmonics, allowing for detailed analysis of continuous margins without relying on predefined landmarks [156]. EFA has been effectively applied to lateral skull photographs, enabling the capture of both cranial and facial morphology for population affinity and sex estimation. Caple et al. [156] demonstrated the utility of EFA, using Principal Component Analysis (PCA) and linear DFA for classification, achieving a 73% accuracy rate in classifying seven groups based on population affinity and sex. EFA has been integrated into practical tools such as SkullProfiler, an R-based software that automates the entire analysis process [155].

#### Imaging techniques

Advancements in medical and engineering technologies have significantly expanded the range of imaging methods used in forensic anthropology [157]. Among these, virtual 3D reconstructions of skeletal remains are increasingly utilized for casework, documentation, and research.

#### Computed tomography

Computed Tomography (CT), initially developed for medical diagnostics, has become a vital non-invasive tool in forensic anthropology, enabling high-resolution 3D reconstructions of skeletal structures. Its growing accessibility, safety, and accuracy have expanded its use across research, education, and diagnostics [157].

CT-based osteological measurements closely align with those from dry bones, typically within 1–3% variance—comparable to standard radiographic margins [158–161]. CT imaging also shows promise in population affinity estimation. For example, Torimitsu et al. [162, 163] achieved classification accuracies of up to 97% using cranial and pelvic morphology from multidetector and postmortem CT scans, combined with machine learning techniques, in Japanese and Western Australian samples.

A persistent limitation in forensic anthropology is the scarcity of comprehensive subadult skeletal collections, often hindered by small sample sizes, preservation issues, and outdated demographic data [164–166]. Virtual CT-based datasets are increasingly leveraged to overcome these constraints by providing standardized and widely accessible reference material [167]. The New Mexico Decedent Image Database (NMDID), for example, includes over 15,000 scans—more than 1,000 of which are subadults—offering diverse skeletal data, though its geographic focus on the American Southwest limits broader applicability and requires cross-disciplinary technical expertise [168]. Similarly, the Bakeng se Afrika (BsA) repository offers over 3,400 micro-CT scans of cranial and postcranial elements from South African populations, enhancing regional representation [169]. The Subadult Virtual Anthropology Database (SVAD) expands global relevance by providing open-access CT and radiographic scans of individuals aged 0–22 from Africa, Asia, and South America, thus improving subadult forensic standards [170].

The integration of CT imaging and virtual skeletal databases is thus playing a transformative role in forensic anthropology, addressing traditional limitations while supporting greater standardization, collaboration, and global applicability.

#### Surface scanning

Surface scanners can capture both texture and colour of skeletal remains, offering insights into surface modifications, taphonomic changes, fractures, and ballistic trauma [157]. However, they are limited to external features and cannot document internal structures or intricate details such as deep grooves. Their accuracy can also be affected by lighting conditions, requiring controlled environments for consistent results [171]. Although not yet widely used in forensic anthropology, studies such as Sholts et al. [172] have shown that 3D scans can accurately capture cranial landmarks for precise measurements. Their affordability, portability, and high resolution make them accessible tools, though the lack of standardised scanning protocols remains a challenge [157].

#### Photogrammetry

Photogrammetry involves capturing multiple overlapping images of an object using digital cameras to create 3D models [173, 174]. Katz and Friess [175] found photogrammetry of human skulls comparable to 3D surface scans for morphological analysis, though its forensic potential remains underexplored [157]. Resources such as the LAMbDA website support its application in forensic anthropology by offering tools to identify cranial landmarks using 3D models, enhancing both accessibility and anatomical accuracy [176].

Oriola et al. [177] employed a cost-effective photogrammetry-based method to reconstruct cranial and mandibular fragments from five Spanish Civil War victims, achieving high anatomical accuracy in the alignment and restoration of the fragmented elements, such as the zygomatic arches with minimal deformation. Virtual models preserved perimortem trauma, and craniofacial measurements closely aligned with traditional methods. DFA identified the continental origin of 87% of 15 known crania; 55% were correctly classified as Spanish, and 27% had high posterior probabilities. Among restored crania, two were classified as Spanish and one as a non-Spanish European. One case yielded inconclusive results, highlighting limits in current reference datasets and European morphological variability. The study demonstrates the promise of photogrammetry in forensic anthropology—particularly for virtual reconstruction and population affinity estimation—while emphasizing the need for expanded databases and improved analytical tools.

#### Magnetic resonance imaging

This imaging technique utilizes nuclear magnetic resonance of excited protons within a strong magnetic field and magnetic field gradients to generate detailed images of both soft tissue and bone [171]. However, magnetic resonance imaging (MRI) is not typically the preferred modality for visualizing bone, as it primarily captures signals from soft tissue, where protons are more abundant, rather than from the denser bone tissue [171]. Despite these limitations, MRI is increasingly being explored in forensic anthropology [178–180]. Several studies have confirmed the accuracy and reliability of long bone measurements obtained from MRI scans, demonstrating that these measurements are comparable to those derived from dry bone elements [178]. MRI has also been used to estimate various biological profile characteristics, such as stature in both sub-adult and adult populations [179, 181] as well as for age estimation research [182, 183]. Additionally, studies have shown that cranial landmarks can be extracted from MRI scans, enabling the collection of craniometric data for the estimation of age and sex [180].

Despite these advances, MRI remains less effective for providing detailed bone structures compared to other imaging modalities [184]. Moreover, while MRI’s potential for estimating population affinity is intriguing, further research is needed to explore its efficacy in this area. The role of MRI in population affinity studies is still underdeveloped, and future investigations should aim to refine its application for forensic anthropology, particularly in relation to skeletal differentiation across populations.

#### X-rays

X-rays produce images by passing electromagnetic radiation through the body, with unabsorbed rays captured on a radiation-sensitive film [185]. Though no longer cutting-edge, radiography remains one of the most widely used imaging tools in forensic anthropology after photography [157]. Radiographs are especially valuable for age estimation in infants and subadults by assessing ossification, epiphyseal fusion, and dental development [186, 187]. Their utility in adults is more limited due to the reliance on skeletal degeneration. Radiography also shows potential for estimating sex, population affinity, and stature, particularly in cases with fleshed remains. For example, costal cartilage ossification has been linked to sex estimation [188]. While many skeletal features—such as the greater sciatic notch or nasal aperture—could be assessed radiographically, reducing the need for invasive methods, this application remains underexplored and would require standardized imaging protocols. Standardized radiographic techniques could also enhance assessments of population affinity by highlighting skeletal traits associated with specific groups [157].

#### Micro-focus X-ray computed tomography

Micro-focus X-ray computed tomography (micro-XCT) is the gold standard for high-resolution, non-invasive imaging of skeletal microstructures, including odonto-skeletal elements. With a resolution of up to 2 μm, micro-XCT employs a microfocus X-ray source, CCD camera, and motorized platform to reconstruct detailed 3D images, facilitating digital preservation for research and education [169, 189]. While its forensic applications remain relatively unexplored, micro-XCT holds significant promise in forensic anthropology due to its capacity to capture both internal and external bone morphologies. The technique enables precise 3D modelling of subtle craniofacial features—such as nasal aperture shape, orbital structure, and dental root morphology—relevant for estimating population affinity. It also reveals internal bone architecture, such as trabecular patterns [174], which may vary across populations due to genetic and environmental factors and offer supplementary metrics for population affinity assessments, particularly when integrated with traditional estimation methods.

Micro-XCT enhances the creation of digital skeletal databases (i.e., BsA repository), especially for underrepresented regions such as South Africa [169]. It is particularly useful for imaging teeth—capturing both internal and external structures such as pulp chambers and root canals—for population affinity analysis. As a non-destructive method, it preserves specimens and enables precise, bias-reduced data collection. However, high costs, long scan times, and limited datasets remain challenges, underscoring the need for broader population-specific references.

**Table 2:** 3D imaging approaches utilised in forensic anthropology

Imaging modality	Mechanism
3D surface scanning	Uses lasers or structured light patterns to capture an object’s shape, dimensions, and surface characteristics, producing higher-resolution images than traditional CT slice thicknesses [157].
CT	Directs collimated X-ray beams through an object, detected by a circular array of photomultiplier tubes [157].
Photogrammetry	Uses a digital camera to capture multiple overlapping images of an object from various angles, documenting its size, shape, position, and orientation, creating a detailed 3D model of the object [173].
MRI	Uses nuclear magnetic resonance of excited protons in a magnetic field to generate images of soft tissue and bone [171]
X-rays	Emits X-ray radiation, which passes through a film, creating an image [185].
micro-XCT	A miniaturized version of X-ray CT, capturing internal structures with 2 μm resolution using a microfocus X-ray source, CCD camera, and motorized platform to reconstruct a 3D image from multiple angles [189].

#### Machine learning

Machine learning (ML) focuses on how computers learn from data [190], using algorithms such as ANNs, decision trees (DTs), RFMs, SVMs, and cluster analysis for classification tasks [191]. These models apply artificial intelligence(AI)-based mathematical techniques to detect patterns and make predictions, excelling at processing large, complex datasets while minimizing human error and saving time [190, 192, 193]. Despite their strengths, limitations in ML applications are related to the availability of powerful computational systems and the technical knowledge required to use them effectively [190, 193].

In forensic anthropology, ML has shown increasing potential, particularly in estimating sex and population affinity using skeletal characteristics as predictors [59]. Morphoscopic traits have proven effective within ML models, offering quantifiable accuracy and reducing observer bias. Hefner and Ousley [101] compared multiple models using cranial MMS traits, as detailed in prior research by Hefner [96] among African, White, and Hispanic Americans, finding that ANNs achieved the highest classification accuracy (88%), followed closely by SVMs and RFMs (around 85%). Nikita and Nikitas [192] evaluated various ML models across six groups, observing that SVMs and linear DFA performed best, while DTs had the lowest accuracy and ANNs required complex parameter tuning. From a different angle, Hefner, Spradley and Anderson [194] investigated Feldesman’s [195] suggestion that DTs could be used when DFA is not feasible due to missing data. They also examined RFMs on combined craniometric and morphoscopic data from European, African, and Hispanic American samples, reporting nearly 90% accuracy—about 4% higher than linear DFA. Their results indicated that both data types offer comparable biological insights, and they advocated for RFMs due to their ability to handle mixed data types effectively [194]. Additionally, tools such as AncesTrees, tested by its developers, showed promising generalization capabilities on unseen data, and Navega et al. [59] concluded that the freely available software holds great promise for future adoption and potential integration into routine forensic practices.

In summary, while the above tools and methods—ranging from traditional morphometric analyses to more recent ML approaches—have demonstrated value in estimating population affinity, their overall accuracy and applicability are contingent on population-specific variation and the availability of representative reference data. The following section explores their application within the uniquely complex South African context, where historical, demographic, and genetic diversity present both challenges and opportunities for forensic anthropology.

## Forensic anthropology in the South African context

South Africa is one of the most demographically diverse countries in the world, with its population comprising approximately 81.4% Black, 8.2% Coloured, 7.3% White, 2.7% Indian or Asian, and 0.4% belonging to other groups (www.statssa.gov.za). This diversity reflects a complex history of migration, settlement, and sociopolitical transformations that have shaped both the genetic and cultural fabric of the nation.

The majority of Black South Africans trace their lineage to Bantu-speaking populations that migrated from Central and West Africa approximately 5,000 years ago. As these groups expanded across sub-Saharan Africa, they diversified into distinct ethnolinguistic communities, including the Sotho, Nguni, Venda, and Shangaan-Tsonga, each with unique cultural traditions and settlement patterns [196–198]. The arrival of European settlers in the 17th century further altered the region’s demographic landscape. The Dutch East India Company established the Cape Colony as a refreshment station for its trade routes, initiating an influx of European settlers—initially Dutch, followed by British, French, and German migrants [196]. South Africa’s Coloured population, a historically and socially defined group, emerged from complex intermixing between enslaved individuals from Malaysia, Indonesia, and India; European settlers; Bantu-speaking groups; and the indigenous Khoisan peoples [199–201]. This diverse genetic heritage has contributed to significant within-group variation [202]. Similarly, the Indian or Asian population traces its origins primarily to South Asia. Although some individuals arrived as enslaved labourers under the Dutch East India Company in the 17th century, the most substantial wave of migration occurred in 1860, when the British colonial administration in Natal (modern-day KwaZulu Natal) recruited indentured labourers from India to work on sugar plantations. Many of these labourers remained in South Africa after their contracts ended, later joined by free migrants in the late 19th and early 20th centuries, further enriching the country’s demographic landscape [203, 204]. These historical migrations and interactions have played a pivotal role in shaping the genetic and cultural diversity of contemporary South Africa. However, the imposition of segregation laws, particularly during apartheid, further influenced the country’s demographic structure, impacting both social organization and biological variation.

The institutionalized racial categorization imposed by apartheid policies had profound consequences on population dynamics and morphological variation in South Africa. The Population Registration Act No. 30 of 1950 classified all South Africans into four racial groups: African, Coloured, Indian/Asian, and White. This classification system, enforced by the 1951 census bureau, reinforced racial segregation, shaping mating patterns and, in turn, contributing to population-level morphological differentiation [15]. Although apartheid officially ended in 1994, these classifications persist in post-apartheid South Africa for redress purposes under the democratic government. Understanding the biological consequences of historical racism offers valuable insights into contemporary population diversity and underscores the lasting effects of institutionalized segregation.

Given South Africa’s demographic complexity, forensic anthropology in the country faces unique challenges, particularly in estimating population affinity. The legacy of apartheid-era racial classification, coupled with the nation’s extensive genetic variation, complicates the application of traditional forensic methodologies. One of the most pressing forensic issues is the high number of unidentified bodies recovered annually in medico-legal facilities [205]. Despite the use of standard identification techniques such as DNA analysis, fingerprinting, and familial matching, numerous individuals remain unidentified due to missing documentation, migration, foreign nationality, and unresolved missing person cases [181]. To address this challenge, the Forensic Anthropology Research Centre (FARC) at the University of Pretoria was established in 2008. In collaboration with the South African Police Service (SAPS), forensic anthropologists at FARC analyse skeletal remains to develop biological profiles, facilitating the identification of unknown individuals. However, the effectiveness of these forensic analyses is hindered by the lack of comprehensive reference datasets, particularly for admixed and underrepresented groups.

In forensic anthropology, population affinity estimation in South Africa is traditionally categorized into three primary groups—Black, Coloured, and White South Africans—based on osteometric cranial and postcranial features [64, 181]. However, government classification standards recognize four groups: African/Black, Coloured, Indian/Asian, and White. A significant limitation in forensic casework is that the South African Forensic Database currently excludes Indian/Asian South Africans due to a lack of reference data and software constraints. This omission limits forensic anthropologists’ ability to accurately represent the country’s full demographic diversity. Additionally, the broad classification of the Black population as a single category fails to account for its extensive genetic and cultural heterogeneity. Despite constituting approximately 81% of the population, this group comprises multiple ethnolinguistic backgrounds shaped by historical events such as the Bantu expansion and colonial-era migrations [52, 198, 206]. The oversimplification of this diversity may compromise forensic identification accuracy.

These population complexities also pose challenges for forensic classification tools such as FORDISC, particularly when applied to heterogeneous populations. The absence of specific reference data for subgroups, such as Indian/Asian South Africans, increases the likelihood of misclassification into broader categories. Moreover, genetic overlap among admixed populations and external factors such as disease or trauma can further contribute to classification errors. While these challenges are not unique to South Africa, they have significant implications for forensic anthropology globally [1, 25, 52, 64, 207]. For instance, Ross et al. [25] demonstrated that Cuban individuals, typically classified as Hispanic in the US, exhibited greater morphological similarity to African American populations and pre-contact Cubans. Similarly, Spradley et al. [24] examined the remains of undocumented migrants who perished while crossing the US-Mexico border and found that their cranial morphology occupied an intermediate position between Native American and White American populations [208]. These findings highlight the necessity of selecting appropriate reference groups when estimating population affinity in forensic contexts. To improve the accuracy of population affinity estimation, it is essential to expand forensic reference datasets [24, 25, 52, 64, 207] and refine analytical tools to accommodate diverse populations [206]. Current research efforts focus on increasing sample diversity and enhancing forensic methodologies to improve classification accuracy. Given South Africa’s unique demographic landscape, these initiatives are crucial for addressing the complexities of forensic identification in heterogeneous populations.

### Methods currently used for estimating population affinity in South Africa

#### Metric approaches

##### FORDISC

To date, custom databases have been developed to facilitate the classification of modern Black, White, and Coloured South Africans for use with the FORDISC program [15, 52]. These databases are frequently employed in medico-legal case analyses [209]. The datasets are derived from 24 cranial and 39 postcranial standard linear measurements collected from the Pretoria Bone Collection at the University of Pretoria, the Raymond A. Dart Collection at the University of the Witwatersrand, and the Kirsten Skeletal Collection at Stellenbosch University. The specimens used span from the late 19th to the mid-20th century. The South African Forensic database for FORDISC currently comprises 141 Black, 177 Coloured, and 109 White South African individuals (Table 3) (G. Krüger, personal communication, 19 April 2024).

**Table 3:** Distribution of reference groups for cranial data from the custom South African database in FORDISC

South African Database			
Reference population	Females	Males	*n*
Black South Africans	52	89	141
Coloured South Africans	62	115	177
White South Africans	49	60	109
**n**	163	264	427

Research on craniometric variation among South African populations has demonstrated the effectiveness of cranial measurements in estimating population affinity, with classification accuracies reaching up to 73% [15, 210]. Postcranial data have shown even greater accuracy, with multivariate approaches achieving classification rates of up to 85% for population affinity and 98% for sex estimation. While FORDISC remains a widely used tool, its reliability is significantly constrained by several factors in the South African context. FORDISC’s effectiveness is hindered by its dependence on reference populations, assumptions of homogeneity, and the underrepresentation of certain groups. These limitations are particularly problematic given South Africa’s complex demographic history and high levels of genetic diversity.

FORDISC classifies individuals based on reference datasets, which may not adequately represent the full spectrum of human variation in South Africa. While South African forensic databases have been developed for Black, White, and Coloured populations [15], they however do not fully encompass the country’s diverse population structure. Indian/Asian South Africans, as well as more recent migrant groups from other parts of Africa, remain absent from these datasets, leading to misclassifications or inconclusive results.

Moreover, variation in postcranial morphology among self-reported South African groups reflects different population histories and selective influences. Decades of positive assortative mating and segregation limited gene flow, maintaining morphological differences among modern South Africans [16]. Black and Coloured populations exhibit higher within-group variation and significant overlap, while White South Africans present more homogeneity and greater between-group differentiation [52]. Intermarriage between historic Khoisan and Bantu-speaking groups [201] has further shaped cranial morphology, particularly among the Xhosa and Zulu [211]. These complexities challenge broad categorizations within FORDISC, as the software may not be able to distinguish subtle but significant morphological distinctions.

A critical limitation of FORDISC is its assumption that reference populations are homogenous, which does not reflect the reality of South African demographics or accurately reflect the biological diversity and substructure present within human populations [54]. Given the distinct ethnic and linguistic groups within the Black South African population, research indicates discernible cranial differences among these groups [198, 206], yet FORDISC often categorizes them under a single broad classification, which can reduce accuracy.

Similarly, the Coloured population is defined by mixed ancestral heritage, incorporating African, European, and Asian lineages [200, 201]. While apartheid-era policies restricted intermarriage between White and “non-White” groups, they were less stringent for Black and Coloured populations [212]. This history has resulted in significant genetic diversity within the Coloured population, further complicating classification efforts. In contrast, White South Africans exhibit lower within-group variation, making them easier to distinguish. However, historical intermixture between European colonists and indigenous or enslaved women [200] introduces additional complexity, challenging rigid ancestral classifications.

South Africa has experienced significant migration, particularly from East and Central Africa, yet these populations are largely absent from forensic reference datasets [213]. The increasing presence of migrant groups further complicates population affinity estimation, as individuals from these backgrounds may not align with any available reference category in FORDISC. This is particularly problematic in forensic casework, where misclassification can impede identification efforts. Indian/Asian South Africans are similarly underrepresented in forensic databases, raising questions about how best to estimate their population affinity. Given that this population has distinct cranial and postcranial characteristics, their absence from reference datasets can lead to significant misclassifications. This omission highlights the need for expanded and updated forensic datasets that accurately represent the full diversity of populations within South Africa. Addressing these challenges requires refining classification methods, expanding forensic databases, and integrating complementary approaches to ensure accurate population affinity estimations in forensic casework.

Beyond cranial and general postcranial metrics, various skeletal elements have been explored for their potential in population affinity estimation. The subpubic angle, for instance, has been identified as a useful discriminator between Black and White South Africans, with notable differences observed between sexes and populations [214]. Similarly, vertebral and sacral measurements have been used to classify individuals from distinct South African groups. Ünlütürk [78] reported a 92% classification accuracy when excluding sex as a variable, with thoracic vertebrae providing the highest accuracy and the sacrum the lowest. When considering all vertebrae and the sacrum together, classification accuracy increased to 98% for differentiating Black and White South Africans. Further, Bidmos et al. [215] assessed metric parameters of the talus, demonstrating population differences with classification accuracies ranging from 80 to 96%. These studies highlight the utility of diverse skeletal elements in refining population affinity estimation within forensic and anthropological contexts in South Africa.

##### Geometric morphometrics

Recent advancements in GMM have led to a reassessment of cranial morphological diversity, even within well-studied populations. This re-evaluation does not challenge the validity of traditional metric and visual approaches, which have achieved classification accuracies exceeding 80–90%, but rather highlights the additional insights that GMM can provide.

In South Africa, studies have employed both traditional craniometric techniques and GMM to investigate cranial variation across different social and “ancestral” groups. One of the earlier applications of GMM in this context was conducted by Franklin et al. [198], who examined cranial variation in 12 modern human populations from southern Africa. Their study included 298 male Bantu-speaking individuals and a small Khoisan sample, analysing 96 cranial landmarks using the shape-analysis software, Morphologika. The findings revealed clear morphological differences between Bantu-speaking and Khoisan individuals. The Khoisan displayed distinctive features such as a pentagonoid cranial vault, a more rounded forehead contour, and a smaller, less prognathic face. While Bantu-speaking populations exhibited close genetic affinities, regional variations were evident. For instance, the crania of southern Bantu groups (e.g., Xhosa, Southern Sotho, and Zulu) were more brachycephalic and less prognathic, likely due to varying degrees of historical admixture with Khoisan groups.

Further supporting the efficacy of GMM, Stull et al. [16] analysed cranial morphology among Black, White, and Coloured South African populations, demonstrating that despite genetic similarities, these groups could be distinguished with high classification accuracies. Traditional craniometric analyses classified Black South Africans with 91% accuracy and Whites with 80%, whereas GMM improved classification accuracies to 93% for Whites and 82% for Coloured individuals. These findings highlight GMM’s ability to capture subtle morphological differences, improving population affinity estimations. Similarly, McDowell et al. [216] examined nasal bone and aperture shape variation among Black, White, and Coloured South Africans using both traditional craniometric variables and GMM, incorporating Generalized Procrustes and elliptical Fourier analyses. Their study [216], which recorded 14 cranial landmarks from 310 skulls found that while all classification accuracies were above chance, the lowest accuracies were observed in the Coloured group. The greatest misclassification occurred between Black and Coloured individuals, likely due to their shared ancestral contributions. The study further noted that misclassification was more common within sex groups than between populations, suggesting that nasal morphology is more strongly influenced by population affinity than by sex.

Maass and Friedling [217] conducted a large-scale GMM study analysing the neurocrania of 774 individuals from South African populations, achieving an overall classification accuracy of 83%. Their study revealed that White South Africans exhibited narrower orbital regions and wider cranial vaults, while Black and Coloured individuals showed closer morphological affinities. DFA identified the most pronounced differences between Black and White groups (mean Mahalanobis distance [MD] = 3.3), with the Coloured group showing intermediate affinities (MD = 1.4 and 2.5, respectively). LOOCV further demonstrated that classification accuracies were highest for Whites (90%), followed by Blacks (82%) and Coloureds (78%).

More recently, Ridel et al. [218] applied a 3D computer-assisted automated landmarking workflow to analyse anatomical variation in 200 CBCT scans. Their study focused on soft and hard tissue structures, including the nasal bones, anterior nasal aperture, zygoma, and maxilla. Results indicated that population affinity accounted for the greatest variation in craniofacial morphology, achieving a 100% classification accuracy. Specifically, White South Africans exhibited longer, more prominent external noses, while Black South Africans had shorter noses with nasal bones more integrated into the skull. Mbonani et al. [219] expanded on this by investigating the influence of population affinity on facial morphology in French and White South African individuals using CBCT scans and GMM. Their study quantified significant morphological differences (*p* < 0.05) in both hard and soft tissue matrices, particularly in ear, eye, and nasal morphology. White South Africans had larger, wider left ears, whereas the French exhibited smaller, narrower ears. Eye shape also varied, with White South Africans displaying narrower eyes compared to the wider eyes of the French group. Nasal morphology differed significantly, with White South Africans having a narrower, more elongated nose, while the French exhibited less pronounced nasal differences. These findings emphasize the importance of population-specific datasets in forensic identification.

Tawha et al. [220] investigated zygomatic shape and size in 158 South African individuals using GMM to assess sex and population affinity. Eight 3D zygomatic landmarks were captured using a Microscribe G2 digitizer, and shape variation was analysed through multivariate regression, discriminant function, and canonical variate analyses. The results indicated significant shape differences among population groups, with Black and Coloured South Africans exhibiting narrower, shorter, and more anteriorly projecting orbital margins, while White South Africans displayed vertically elongated and recessed orbital margins. The greatest morphological distinction was observed between White and Black South Africans, with classification accuracies improving when population and sex were combined. Pairwise comparisons showed that Black South Africans were distinguishable from Whites with accuracies of 76% (females) and 77% (males), while Coloured individuals exhibited more variability due to their diverse ancestral contributions, including Khoisan, Bantu-speaking, European, and Asian lineages. The study suggested that the observed zygomatic variations align with historical thermoregulatory adaptations, with African-descended populations exhibiting features suited to warmer climates, while European-descended individuals displayed traits associated with colder environments [221]. These findings reinforce the utility of zygomatic morphology in forensic applications but highlight the need for population-specific standards for South African groups with shared African descent.

Investigations into intra-population variation have also revealed significant morphological differences among Black South African socio-cultural identity groups. Sapo [222] analysed cranial variation among Swazi and Sotho groups, identifying distinct cranial traits that differentiate these populations. Building on this, Ridel and L’Abbé [206] employed 3D imaging techniques to explore the influence of socio-cultural identity on midfacial morphology among modern Black South Africans. Their study used parametric and non-parametric tests to accommodate multivariate normality deviations, revealing significant shape variations across linguistic and cultural lineages. Classification accuracies ranged from 73 to 94%, with Ndebele, Zulu, Tswana, and Swati groups displaying distinct midfacial characteristics compared to Sotho and Tsonga groups. Morphological differences were consistent across various shape configurations, underscoring the advantages of 3D GMM in maintaining object geometry and capturing fine-scale morphological variation. Despite its strengths, the study acknowledged limitations related to sample size and the need for broader datasets to refine analyses further, particularly regarding sex- and age-related variation.

Collectively, these studies highlight the complex interplay of genetic, ecological, and historical factors in shaping cranial and facial morphology among South African populations. The observed variations are influenced by a combination of ecogeographical adaptations, population history, and socio-cultural factors. From a forensic perspective, recognizing population-specific morphological traits enhances identification accuracy, allowing for more precise estimations of population affinity, even in highly diverse populations. However, a notable limitation of current research is the lack of GMM applications to postcranial elements. Given the established success of GMM in cranial and facial analyses, future research should explore its potential in postcranial morphology to provide a more comprehensive understanding of population affinity and skeletal variation in forensic contexts.

#### Non-metric approaches

##### Macromorphocopic traits

Research on non-metric traits in South Africa remains limited [209]. Historically, forensic anthropologists in the region have relied on outdated methodologies, such as those proposed by De Villiers [223] and international standards established by Krogman [224]. These approaches were largely typological, lacked population-specific data, and failed to account for the full range of human variation [223, 224]. A significant advancement came with the work of L’Abbé et al. [15], who analysed 13 MMS traits in modern Black, White, and Coloured South Africans. Their study incorporated nine traits from Hefner [96], two from Bass [225], and two from Hauser and De Stefano [95]. These traits were selected based on their demonstrated correlation with population affinity in North American populations and their frequent use in South African forensic casework, particularly those related to the nasal and maxillary regions. While L’Abbé et al. [15] provided valuable insights into trait distributions across population groups—reflecting the inherent heterogeneity of the South African population—they did not develop classification models, leaving the forensic applicability of MMS traits in South Africa uncertain.

To date, forensic anthropologists in South Africa predominantly rely on metric methods to estimate population affinity from skeletal remains [226]. Although non-metric methods are available, they remain under-researched and insufficiently validated for forensic application in the local context. Addressing this gap, recent research by Liebenberg [209] evaluated the effectiveness of MMS traits in combination with traditional craniometric measurements. The study assessed 17 MMS traits and 25 standard linear cranial measurements from a sample of 660 individuals representing Black, White, and Coloured South Africans. Statistical analyses, including Kruskal-Wallis and Dunn’s post hoc tests, revealed significant population differences in 13 of the 17 traits. However, no single trait was exclusive to any one population group. RFM demonstrated that MMS traits classified population affinity with 79% accuracy—comparable to traditional craniometric methods. The most informative traits were those of the nasal region, including the inferior nasal margin, nasal bone contour, and nasal aperture shape. This study represents the first comprehensive analysis of MMS trait variation and classification performance in a contemporary South African sample, validating their potential forensic utility. Notably, Liebenberg’s [209] findings align with previous research [15, 16, 118], reaffirming the substantial cranial overlap among South African population groups. Misclassification patterns were consistent across both MMS and craniometric datasets, with Coloured South Africans frequently misclassified with either Black or White South Africans [209, 226]. However, Black and White South Africans were rarely misclassified as one another. These results support the findings of Hefner, Spradley, and Anderson [194], who demonstrated that both craniometric and MMS data provide comparable insights into population relationships. Further refinement of MMS-based classification models will require investigations into factors such as sexual dimorphism, age-related changes, edentulism, and asymmetry. Importantly, the integration of MMS traits with craniometrics yielded the highest classification accuracy (81%), surpassing metric-only models (72%). This suggests that MMS traits capture both size and shape variation more effectively than linear measurements alone, reinforcing their value in forensic anthropological practice [209, 226]. Despite concerns regarding the repeatability of certain traits, this research contributes to the global MaMD and represents a critical step toward enhancing population affinity estimations in South African forensic casework.

While cranial MMS traits have demonstrated potential in forensic contexts, population overlap and morphological variation within and between groups remain complex and difficult to quantify. A comprehensive biological profile requires the exploration and validation of multiple methodological approaches. One notable gap in the field is the absence of validated databases for postcranial MMS traits, raising questions about their forensic applicability. To address this, Bothma et al. [227] conducted the first assessment of postcranial MMS traits in a modern South African population using the methodology proposed by Spiros [130]. Bothma et al. [227] examined 11 postcranial MMS traits in 271 individuals of Black, White, and Coloured South African descent. Inter- and intra-observer reliability varied from fair to almost perfect, with the exception of the accessory transverse foramen of C1, which exhibited poor agreement between observers. Seven of the 11 traits showed statistically significant differences between at least two of the population groups. Univariate and multivariate RFM models were constructed to evaluate classification accuracy, with univariate models achieving accuracies between 33% and 53%, while multivariate models performed slightly better at 55–62%. Variable importance rankings indicated that traits associated with spinous process bifurcation were the most discriminatory. However, the study concluded that postcranial MMS traits do not outperform existing methods for estimating population affinity in South Africa. The findings underscore the need for further research to enhance the forensic applicability of postcranial MMS traits, particularly in the development of population-specific databases.

These studies underscore the potential of MMS traits in South African forensic anthropology, particularly when integrated with traditional metric approaches. However, the findings also highlight the persistent challenges of population overlap and intra-group variation. To improve classification accuracy and forensic applicability, future research should explore the combined use of cranial and postcranial MMS traits in population affinity estimation. Expanding the database of validated traits and refining trait selection criteria will be essential to strengthening the reliability and repeatability of MMS-based methods in forensic casework.

#### Combined metric-macromorphoscopic approaches

McDowell et al. [228] investigated nasal aperture size and shape variation in Black and White South Africans by integrating morphoscopic and metric analyses. Their study digitized 13 landmarks from the bony nasal region of 152 crania using an electromechanical instrument to conduct both GMM and craniometric analyses. EFA was employed to assess nasal aperture shape by analysing photographic outlines. PCA and DFA were applied to identify patterns of variation and classify individuals. The classification accuracy for Black South Africans ranged from 95 to 96%, while White South Africans were correctly classified with an accuracy of 91–94%. When considering both sex and population affinity in a four-way classification, accuracy declined to 56–70%, with most misclassifications occurring between males and females within each group. This suggests a lack of significant sexual dimorphism in nasal aperture shape. Overall, the study demonstrated that quantifiable differences in nasal aperture shape exist between Black and White South African populations across all statistical methods employed.

Expanding on this research, Dinkele [229] examined mid-craniofacial shape and size variation among Black, White, and Coloured South Africans using a combination of metric, non-metric, and GMM assessments. The study analysed 392 crania from South African skeletal collections, focusing on the orbital, nasal, zygomatic, and maxillary regions in both 2D and 3D contexts. Univariate and multivariate statistical analyses were conducted to characterize variation and estimate population affinity. The findings suggested that mid-craniofacial variation is tightly integrated across these anatomical regions due to functional, regional, and developmental proximities. Population-specific trends emerged in the analysis, with Black South Africans exhibiting wider and shorter midfacial regions compared to White South Africans, who had the narrowest orbital, zygomatic, and nasal breadths along with the longest upper facial, orbital, and nasal heights. White South Africans also displayed inferiorly angled orbits, elongated nasal apertures, and anteriorly projecting nasal bridges. In contrast, Black South Africans exhibited rounder nasal apertures, less anteriorly projecting nasal bridges, and more anteriorly projecting maxillary regions. Coloured South Africans demonstrated substantial craniofacial heterogeneity, which contributed to lower population affinity classification accuracies. Notably, the nasal and maxillary regions exhibited the greatest population-specific variation. In terms of methodological performance, 2D metric methods yielded the lowest classification accuracies (27–60%), followed by non-metric assessments (57–82%). In contrast, metric and GMM assessments demonstrated high repeatability (≥ 95%), suggesting greater reliability for forensic applications. GMM shape analyses produced the highest classification accuracies (75–98%), highlighting the presence of 3D shape differences between South African population groups. These findings reinforce the existence of a continuum of ancestral variation with significant overlap among South African populations, emphasizing the need for multivariate classification standards to improve population affinity estimation.

Both studies highlight the advantages of integrating multiple methodological approaches in forensic anthropology. While individual methods—whether metric, non-metric, or GMM—offer valuable insights, each has inherent limitations. Metric and non-metric methods alone may be affected by observer subjectivity and measurement inconsistencies, while GMM, though highly repeatable, requires specialized expertise and equipment. By combining multiple techniques, forensic anthropologists can capitalize on their respective strengths, increasing classification accuracy and improving the robustness of population affinity estimations. The integration of metric and non-metric data, alongside GMM approaches, provides a more holistic understanding of human variation. This multidimensional approach enables forensic practitioners to navigate South Africa’s complex demographic landscape more effectively, particularly in contexts where misclassification risks are heightened due to overlapping ancestral traits. Furthermore, the successful application of combined methodologies in South African forensic casework could contribute to the development of more inclusive and population-specific forensic standards, ultimately improving the reliability of identifications in both forensic and bioarchaeological contexts.

Despite these advancements, this still highlights the fact that research on postcranial non-metric traits remains limited in the South African context. This suggests that further investigation is needed to determine whether postcranial traits, particularly when analysed using combined methods, can offer additional discriminatory power. Given the demonstrated effectiveness of integrating metric, non-metric, and GMM techniques in craniofacial analyses, future research should extend these approaches to postcranial skeletal elements. The inclusion of GMM in postcranial assessments, which has not yet been thoroughly explored, may enhance classification accuracy and provide new insights into population-specific morphological variation.

Overall, these findings underscore the importance of a comprehensive, multivariate approach to population affinity estimation. Future studies should focus on refining classification models through the integration of cranial and postcranial data while further evaluating the applicability of advanced morphometric techniques in forensic contexts.

#### MaMD analytical tool

It is worth noting, while the MaMD analytical tool has not yet undergone specific validation for South African populations, its application in this context presents a compelling opportunity to enhance forensic anthropological analyses. Given the limitations of existing methods such as FORDISC, MaMD’s capacity for multivariate analysis enables the simultaneous evaluation of both metric and MMS traits, offering a more comprehensive framework for estimating population affinity and variation [125]. This capability is particularly relevant in South Africa, where conventional classification tools often struggle to account for the country’s extensive genetic diversity. South Africa’s population exhibits significant biological complexity, shaped by a dynamic interplay of indigenous populations, historical migration patterns, and extensive gene flow. Traditional single-trait classification approaches may not fully capture this diversity, whereas MaMD’s multidimensional methodology has the potential to offer a more refined understanding of population structure. A key advantage of MaMD lies in its adaptability, as it allows for the integration of data from diverse populations—an attribute already demonstrated in its existing dataset [125]. Currently, the MaMD analytical tool incorporates data from over 8,000 individuals, including South African populations [125]. This extensive and heterogeneous dataset provides a valuable foundation for studies involving South African demographic groups, including the Nguni and Sotho-Tswana subgroups in Black South Africans, as well as Khoisan, European-descendant, and Coloured populations. The tool’s capacity for customization ensures that it can be adapted to reflect the unique genetic and phenotypic characteristics of these groups, thereby enhancing the precision of population affinity estimations. Additionally, its integration of both metric and non-metric traits, a feature that is particularly beneficial in regions with intricate demographic histories. This holistic approach facilitates a more comprehensive assessment of population variation and affinity. While empirical validation of MaMD’s performance in South African forensic contexts remains a future priority, its demonstrated efficacy in analysing genetically diverse populations underscores its potential as an invaluable tool for advancing forensic anthropological research in the region.

## Discussion and conclusion

Population affinity estimation is a crucial aspect of forensic anthropology, providing valuable information for identifying unknown individuals. However, in South Africa, this process is particularly challenging due to the country’s complex population history, genetic diversity, and sociopolitical influences. The forensic landscape in South Africa has been shaped by centuries of migration, colonial rule, and apartheid-era racial classifications, all of which have influenced both biological variation and the frameworks used to categorize individuals. Forensic anthropologists must not only acknowledge these complexities but also develop methodologies that are both scientifically rigorous and locally relevant.

Traditional population affinity estimation methods have relied heavily on metric and nonmetric analyses of skeletal remains. However, the limitations of traditional forensic methodologies are particularly evident in the application of tools such as FORDISC, which, despite its widespread use, lacks adequate reference samples for South African populations. This shortfall contributes to misclassifications, especially for admixed or historically marginalized groups, such as the Coloured and Indian/Asian South African populations, whose morphological diversity is poorly represented in skeletal collections. The reliance on North American standards for forensic analyses further exacerbates these challenges, as population differences between North American and South African groups have been well-documented.

In response to these challenges, forensic anthropologists have increasingly turned to advanced methodologies that leverage new technologies to improve classification accuracy. GMM, ML, and 3D imaging have emerged as powerful tools for forensic analysis. These techniques enable researchers to capture subtle morphological differences that traditional metric and non-metric methods may overlook. For example, GMM allows for the precise quantification of craniofacial shape variations, while ML models can analyse large datasets and detect complex patterns that human observers might miss. The combination of these methods has the potential to improve forensic classification accuracy by reducing observer bias and providing a more objective approach to population affinity estimation.

Despite these advancements, there remain significant gaps in forensic anthropology in South Africa. One major limitation is the continued focus on cranial traits at the expense of postcranial skeletal elements. While cranial morphology has traditionally been the primary focus of population affinity estimation, postcranial bones also exhibit population-specific variations that could enhance classification accuracy. Research on postcranial non-metric traits in South Africa is still in its early stages, and further studies are needed to determine how these traits can be incorporated into forensic methodologies. Additionally, while ML and AI-based classification models show great promise, they require extensive validation to ensure their accuracy and applicability to diverse populations. Ethical considerations must also be addressed, particularly regarding data privacy, consent, and the potential biases that may be embedded in algorithmic models.

Another challenge is the classification framework currently used in South African forensic anthropology. Many forensic reports continue to categorize individuals into broad racial or population groups—Black, White, and Coloured—without considering the extensive variation within these categories. For instance, the Black South African population consists of multiple ethnolinguistic groups, such as the Nguni (Zulu, Xhosa, Swazi, and Ndebele) and Sotho-Tswana (Sotho, Tswana, and Pedi), each of which may exhibit distinct skeletal characteristics. Similarly, the Coloured population, due to its complex mixed heritage, does not fit neatly into a single morphological profile. The continued use of broad classifications fails to reflect this diversity and can lead to misleading forensic conclusions.

## Future considerations and recommendations

To enhance forensic anthropology in South Africa and improve population affinity estimation, several critical areas require development. Expanding and diversifying reference datasets is a top priority. Existing skeletal collections, such as the Pretoria Bone Collection and the Raymond A. Dart Collection, offer valuable resources but remain limited in size, geographic scope, and temporal relevance. New and inclusive repositories—such as the BsA digital skeletal repository—are essential for reflecting contemporary population variation influenced by environmental, nutritional, and genetic factors.

Incorporating postcranial elements into affinity estimation represents another important step forward. While cranial features are well-studied, postcranial bones such as the femur, pelvis, and tibia show population-specific variation that, when analysed through GMM, could improve classification accuracy. Standardized protocols and further research into how these traits reflect genetic or historical patterns in South Africa are needed. Additionally, the use of ML and AI holds significant potential for automating classification, identifying subtle patterns, and handling large datasets. However, to ensure fairness and transparency, ML models must be trained on representative South African samples, and their outputs should be validated against traditional methods.

Progress also depends on investments in training and infrastructure. Establishing specialized forensic anthropology programs, promoting interdisciplinary collaboration, and supporting ongoing professional development through workshops and conferences will help build local capacity and ensure methodological rigor.

Ethical responsibility is equally vital. Given South Africa’s legacy of racialized science, forensic practices must be culturally sensitive, community-engaged, and committed to avoiding outdated racial constructs. Transparency and community consultation should guide research and application.

By addressing these priorities—diverse reference datasets, technological integration, methodological refinement, capacity building, and ethical engagement—South Africa can establish a globally relevant model for scientifically and ethically grounded forensic anthropology.

## Data Availability

Data sharing not applicable to this review article as no datasets were generated or analysed during the current study.
